# A Novel and Practical Chromatographic “Fingerprint-ROC-SVM” Strategy Applied to Quality Analysis of Traditional Chinese Medicine Injections: Using KuDieZi Injection as a Case Study

**DOI:** 10.3390/molecules22071237

**Published:** 2017-07-23

**Authors:** Bin Yang, Yuan Wang, Lanlan Shan, Jingtao Zou, Yuanyuan Wu, Feifan Yang, Yani Zhang, Yubo Li, Yanjun Zhang

**Affiliations:** 1 College of Traditional Chinese Materia Medica, Tianjin University of Traditional Chinese Medicine, 312 Anshan West Road, Tianjin 300193, China; yang3023008@163.com (B.Y.); wangyuan0041@163.com (Yuan W.); shanlanlan12@163.com (L.S.); 15620935753@163.com (Yuanyuan W.); feifanyang163@163.com (F.Y.); 15202209631@163.com (Yani Z.); 2 Tonghua Huaxia Pharmaceutical Co., Ltd., 3333 Tuanjie Road, Tonghua 134100, China; hxyyzl@126.com; 3 Tianjin State Key Laboratory of Modern Chinese Medicine, Tianjin University of Traditional Chinese Medicine, 312 Anshan West Road, Tianjin 300193, China

**Keywords:** chromatographic fingerprint, receiver operating characteristic curve, support vector machine, traditional Chinese medicine injections, quality analysis

## Abstract

Fingerprinting is widely and commonly used in the quality control of traditional Chinese medicine (TCM) injections. However, current studies informed that the fingerprint similarity evaluation was less sensitive and easily generated false positive results. For this reason, a novel and practical chromatographic “Fingerprint-ROC-SVM” strategy was established by using KuDieZi (KDZ) injection as a case study in the present article. Firstly, the chromatographic fingerprints of KDZ injection were obtained by UPLC and the common characteristic peaks were identified with UPLC/Q-TOF-MS under the same chromatographic conditions. Then, the receiver operating characteristic (ROC) curve was used to optimize common characteristic peaks by the AUCs value greater than 0.7. Finally, a support vector machine (SVM) model, with the accuracy of 97.06%, was established by the optimized characteristic peaks and applied to monitor the quality of KDZ injection. As a result, the established model could sensitively and accurately distinguish the qualified products (QPs) with the unqualified products (UPs), high-temperature processed samples (HTPs) and high-illumination processed samples (HIPs) of KDZ injection, and the prediction accuracy was 100.00%, 93.75% and 100.00%, respectively. Furthermore, through the comparison with other chemometrics methods, the superiority of the novel analytical strategy was more prominent. It indicated that the novel and practical chromatographic “Fingerprint-ROC-SVM” strategy could be further applied to facilitate the development of the quality analysis of TCM injections.

## 1. Introduction

Traditional Chinese medicine (TCM) injections had been regarded as a “double-edge sword” in recent years [1], because they cand provide quick efficacy, but have dramatic safety risks. Until now, the epidemic exploration has indicated that the adverse drug reactions (ADRs) of TCM injections occur frequently, which account for over 70% ADRs of TCM [2]. For the purpose of improving the safety of TCM injections, re-evaluation the safety of TCM injections (RESTI) was prescribed by the China Food and Drug Administration (CFDA). As the quality of TCM injections is the key guarantee of safety, the quality control of TCM injections is an extremely important component of RESTI. Meanwhile, it is well known that the storage condition of TCM injections is much more stringent than other TCM preparations, which indicates that the stability of quality is closely related to the safety. Therefore, establishing a rapid and practical method to monitor the quality is an inevitable requirement of improving clinical safety of TCM injections.

As a comprehensive and non-targeted analysis technology, fingerprint analysis of TCM represents a comprehensive qualitative approach to species authentication and quality evaluation [3,4,5,6]. In 1991, the World Health Organization (WHO) accepted chromatographic fingerprint technology as an identification and qualification technique for medicinal herbs [7]. Then, in 2000, CFDA officially issued a guideline for the fingerprint establishment of TCM injections and all the TCM injections were required to be standardized by chromatographic fingerprints [8]. Besides the CFDA, the Food and Drug Administration (FDA) [9] and European Medicines Evaluation Agency (EMEA) [10] accepted fingerprints to control the quality of herb products. With the wide application in quality control, the similarity of fingerprints, based on calculating the correlative coefficient of chromatograms, is generally used to evaluate the quality of TCM injections.

**Figure 1:**
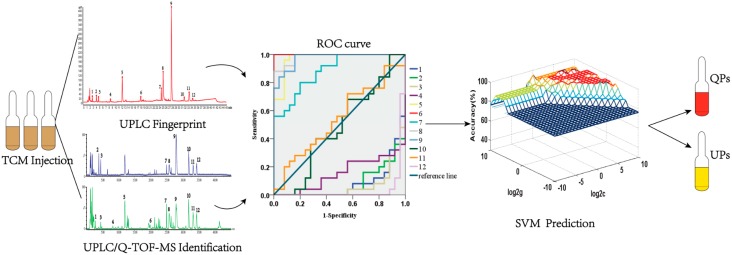
The flow diagram of the chromatographic “Fingerprint-ROC-SVM” strategy.

However, fingerprint similarity evaluation is a qualitative evaluation method, which depends on the distribution ratio of chemical components. It is not sensitive enough to evaluate the quality deviation caused by the differences of component contents. In addition, some researches indicated that it could misjudge the unqualified products as qualified. Recently, principal component analysis (PCA) or hierarchical clustering analysis (HCA) was applied to improve the accuracy of fingerprint analysis [11,12,13,14]. While PCA and HCA are widely used as a classification method, neither can directly predict the quality of independent unknown samples. Therefore, a novel and practical chromatographic “Fingerprint-ROC-SVM” strategy (Figure 1) was established in this article, which makes up for the limitations of fingerprint analysis and also provides a novel and practical analytical strategy to facilitate the development of the quality analysis of TCM injections.

## 2. Results and Discussion

### 2.1. Fingerprints and Similarity Evaluation

The UPLC chromatographic conditions were optimized to obtain a wealth of chromatographic information and good separation effect of the fingerprint. In the optimization, the mobile phase, flow rate, column temperature and the wavelength were examined and are described in the Supplementary File 1. Then, the developed chromatography method was validated by the repeatability, precision, and stability. The results described in Supplementary File 1 indicated that the chromatography method for the fingerprint analysis of KuDieZi (KDZ) injection is reliable and applicable.

**Figure 2:**
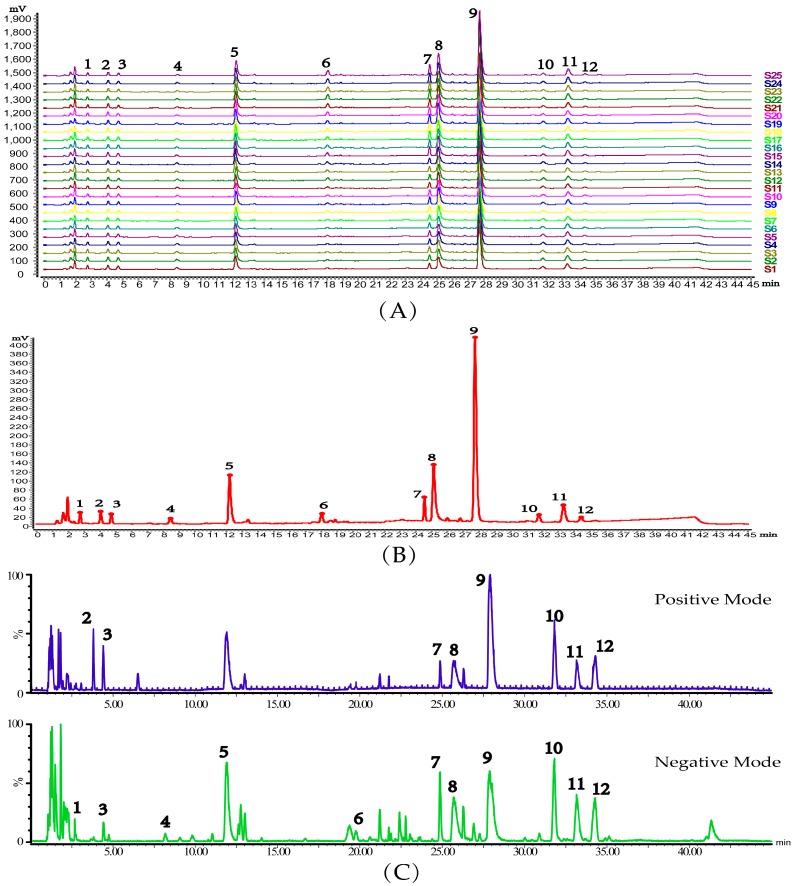
Chromatogram profiles of (**A**) UPLC fingerprints of 25 batches of KDZ injection after peak alignment; (**B**) The reference fingerprint of KDZ injection showing 12 common peaks originating from the SESCFTCM and (**C**) The UPLC/Q-TOF-MS BPI chromatograms of KDZ injection in both positive and negative mode.

After the chromatography method was established, the 25 batches of qualified products (QPs) were analyzed under the optimized chromatographic conditions, and the fingerprints of the 25 batches of QPs were obtained. All the chromatograms were matched by the Similarity Evaluation System for Chromatographic Fingerprint of Traditional Chinese Medicine (SESCFTCM, Version 2.0, Beijing, Chinese Pharmacopoeia commission, Figure 2A). A total of 12 peaks (from peak 1 to 12), shared by all the chromatograms and covered more than 90% of the total area, were assigned as characteristic peaks. Then, the reference fingerprint of KDZ injection was obtained by matching the 10 batches of QPs chromatograms with the SESCFTCM (Figure 2B). In addition, the characteristic peaks were identified by UPLC/Q-TOF-MS with the same UPLC chromatographic conditions. As a result, the base peak intensity (BPI) chromatograms of QPs in both positive and negative mode are shown in Figure 2C, and the characteristic peaks were identified with the exact relative molecular weight and fragment ions compared with reference standards and the information described in references (Table 1).

Based on the fingerprints and the 12 identified characteristic peaks, the similarity values of all the samples were calculated using the SESCFTCM. Similarities were calculated by comparing 25 batches of QPs fingerprints with the reference fingerprint, and the similarity values are presented in Table S1 in supplementary material. Meanwhile, the fingerprints of 25 batches of unqualified products (UPs) were recorded by the same method (Figure S1), and the similarities of each sample to the reference fingerprint were calculated as the method described above (Table S1). The results indicated that the similarities of QPs and UPs to reference fingerprint were all higher than 0.9, which illustrated that the fingerprint similarity could not correctly distinguish QPs with UPs. In other words, if the fingerprint similarity ias used to control the quality of KDZ injection, it easily generates false positive results that identify the unqualified samples as qualified samples. At present, the most widely used method for the similarity evaluation of fingerprint is the cosine method. If the fingerprint was set to vector A (A_1_, A_2_, A_3_,…, A*_n_*) and the reference fingerprint set to vector B (B_1_, B_2_, B_3_,…, B*_n_*), it can be seen from the Equation (1) that the similarity value could be obtained by calculating the cosine value of A and B. However, the similarity calculated by cosine is only sensitive to the relative proportions between the compositions of the sample. Similarity calculated by cosine is a qualitative similarity in the distribution ratio and is not sensitive to the change in the concentration of the constituents. When the peak area distribution is wide, the methods are not sensitive to the difference of data. Therefore, the similarity change caused by the change of the characteristic peaks content in the fingerprint could not be accurately evaluated.

(1) $$S_{f}=cos\theta =\frac{A\cdot B}{\left|A\right|\times \left|B\right|}=\frac{\overset{n}{\underset{i=1}{\sum }}A_{i}B_{i}}{\sqrt{\overset{n}{\underset{i=1}{\sum }}A_{i}{}^{2}}\times \sqrt{\overset{n}{\underset{i=1}{\sum }}B_{i}{}^{2}}}$$

### 2.2. The Establishment of “Fingerprint-ROC-SVM” Prediction Model

**Figure 3:**
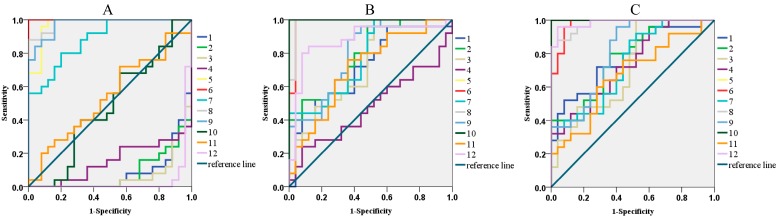
The ROC curve to optimized the specific characteristic peaks. (**A**) The QPs vs. UPs group; (**B**) The QPs vs. HTPs group; (**C**) The QPs vs. HIPs group.

In order to improve the accuracy of the evaluation in quality control, a novel “Fingerprint-ROC-SVM” prediction model was established. On the basis of the identified characteristic peaks, the receiver operating characteristic (ROC) curves were used to optimize the characteristic peaks with more sensitivity and specificity, because the sensitivity and specificity corresponded to the true positive rate and the false positive rate [18]. In the study, QPs samples were regarded as the control groups, and the UPs samples were regarded as test groups. Then, ROC curves and binary logistic regression were used to obtain the AUCs of the 12 characteristic peaks. The characteristic peaks are relatively exclusive when the AUC value is greater than 0.7 [19,20]. As a result, the AUCs of five characteristic peaks are greater than 0.7, and the AUCs distributed 0.89–0.98 at a 95% confidence interval (Figure 3A).

**Figure 4:**
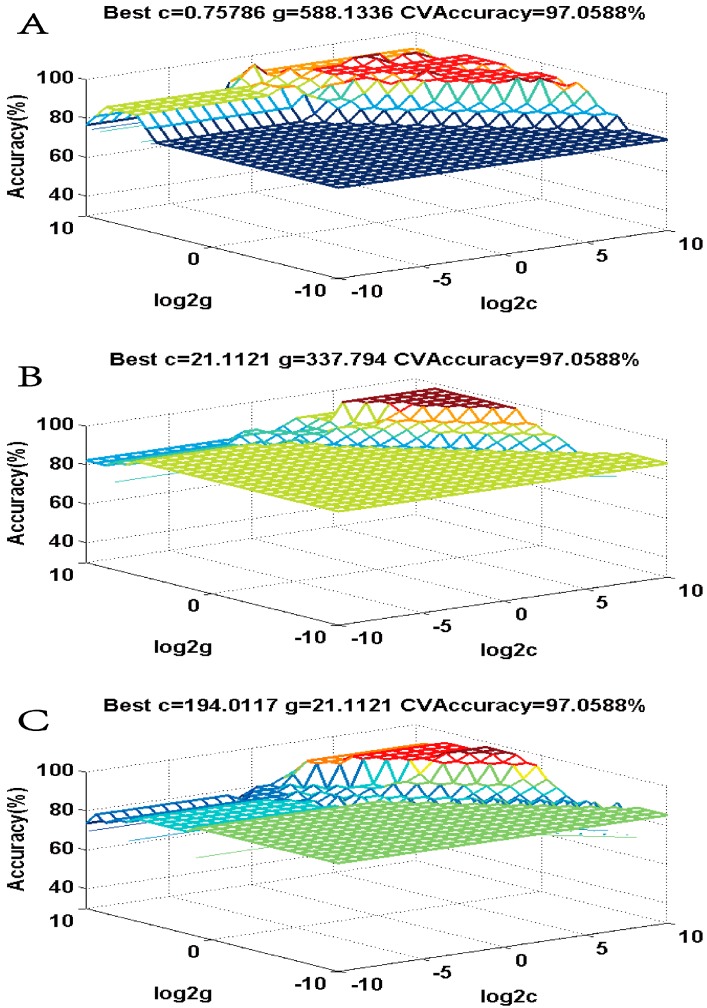
Three-dimensional view of the SVM model of the optimized characteristic peaks. (**A**) The QPs vs. UPs group; (**B**) The QPs vs. HTPs group; (**C**) The QPs vs. HIPs group.

After the five characteristic peaks were optimized, we established the support vector machine (SVM) model of QPs and UPs to monitor the quality of KDZ injection. The peak areas of the specific characteristic peaks in these groups were used to build the SVM models. Two-thirds of the selected data were used as the training set, and the remaining data were used as the test set. The model was processed to establish how to classify the unqualified samples as qualified samples. The training set was established for the classified model, and the test set was employed to obtain the accuracy of the SVM model [21]. The cross-validation accuracy of the model showed good prediction performance and the parameters Best c, Best g, and CV accuracy of the cross-validation method were 0.76, 588.13, and 97.06%, respectively (Figure 4A). The high accuracy of the results demonstrated that the SVM model built by the five characteristic peaks is sensitive and accurate, thus it could be used to monitor the quality of KDZ injection.

### 2.3. Application of the Novel Analytical Strategy

**Figure 5:**
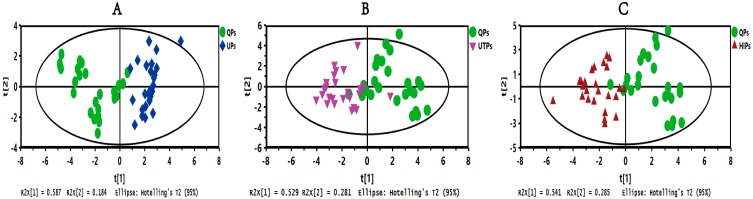
Score scatter plots from the PCA model in differentiating the KDZ injection samples. (**A**) The QPs vs. UPs group; (**B**) The QPs vs. HTPs group; (**C**) The QPs vs. HIPs group.

As described above, we established a reliable “Fingerprint-ROC-SVM” prediction model, whose classification and prediction ability could be used to monitor the quality of KDZ injection. As mentioned, the established prediction model was successfully used to classify the qualified and unqualified KDZ injection. The predication accuracy reached 100.00%, which indicated that the UPs could be distinguished with the QPs correctly. Furthermore, to make the advantage of the novel analytical strategy more evidently, the PCA and HCA were performed as comparisons. PCA was performed on SIMCA-P 13.0 software (Umetrics AB, Umea, Sweden) and all 12 common characteristic peaks of 25 batches of QPs and UPs samples were used to draw a score scatter plot, which reflects the degree of dispersion between the samples. As shown in Figure 5A, it is obvious that there was not a complete separation between QPs and UPs samples, which indicated that the PCA could not classify the qualified and unqualified KDZ injection accurately. The HCA was performed on Predictive Analytics Software (PASW) 18.0 (IBM, Chicago, IL, USA) and the results indicated that HCA could not classify the qualified and unqualified KDZ injection accurately either (Figure S2A). PCA and HCA are widely used as classification methods, however, they could not predict independent unknown samples directly. A comparative study of the “Fingerprint-ROC-SVM” prediction model between PCA and HCA indicated that the established prediction model based on the combination of ROC and SVM could effectively and accurately monitor or predict the quality of KDZ injection.

In order to further verify the feasibility of the novel analytical strategy, the high-temperature processed samples (HTPs) and high-illumination processed samples (HIPs) samples of KDZ injection were used. Using the same procedure described above, after the fingerprints of HTPs and HIPs were obtained, fingerprint similarity evaluation was performed. The results, shown in Figures S3 and S4 and Table S1 in Supplementary File 2, illustrated that the similarity evaluation could not distinguish the QPs with HTPs and HIPs accurately either. Then, the ROC curve was applied to optimize the characteristic peaks with more sensitivity and specificity. As shown in Figure 3B,C, there were 11 and 12 characteristic peaks of HTPs and HIPs, and the AUCs, which greater than 0.7, were optimized through comparison with QPs. Then, the peak areas of the specific characteristic peaks in each group were used to establish the SVM models using the same method described above (Figure 4B,C). The cross-validation accuracy of each model showed good prediction performance, and the prediction accuracy reached 93.75% and 100.00%, respectively. In addition, PCA and HCA were also used as contrasts, and the results indicated that PCA (Figure 5B,C) and HCA (Figure S2B,C) could not classify the QPs with HTPs and HIPs of KDZ injection accurately. The comparative study once again demonstrated that the novel analytical strategy could accurately separate the QPs with the HTPs and HIPs of KDZ injection. Therefore, it could effectively and accurately monitor or predict the quality of KDZ injection.

## 3. Materials and Methods

### 3.1. Materials and Reagents

Reference standards of uridine, guanosine and apigenin-7-*O*-β-d-glucopyranoside were purchased from Tianjin WanXiangHengYuan Technology Co., Ltd. (Tianjin, China). Caftaric acid was purchased from Shanghai Yuanye Bio-Technology Co., Ltd. (Shanghai, China). Adenosine, chlorogenic acid and cichoric acid were obtained from National Institutes for Food and Drug Control (Beijing, China). Luteolin-7-*O*-β-d-glucopyranoside was provided by Tonghua China Pharmaceutical Co., Ltd. (Jilin, China). The purity of all standards was determined to be more than 98% by normalization of the peak areas detected by HPLC-DAD. HPLC-grade acetonitrile and formic acid were purchased from Merck (Darmstadt, Germany) and Concord Science and Technology Co., Ltd. (Tianjin, China), respectively. Distilled water was obtained from Watson (Guangzhou, China). All other reagents were of analytical grade.

### 3.2. Sample Collection and Preparation

A total of 50 batches of KDZ injection were provided by Jilin Tonghua China Pharmaceutical Co., Ltd. (Jilin, China). Among them, 25 batches of KDZ injection were QPs and the other 25 batches of KDZ injection, beyond the expiry date, were UPs. A 4 mL of each batch of QPs and UPs was filtered through the microporous membrane (0.22 μm) before direct injection analysis, regarded as QPs samples (QP1-QP25) and UPs samples (UP1-UP25). Moreover, the other 4 mL of each batch of QPs was divided into two parts and then processed under high temperature and high illumination intensity, respectively. The HTPs (HTP1-HTP25) were stored at 40 °C under the condition of 30% RH and 0 lx; while, the HIPs (HIP1-HIP25) were stored at 25 °C under the condition of 30% RH and 5000 lx. Both the HTPs and HIPs were collected after ten days and filtered through the microporous membrane (0.22 μm) for direct analysis.

In addition, the standards of uridine, adenosine and guanosine were weighed accurately and prepared in water at a final concentration of 25.20, 22.20 and 23.40 μg/mL, respectively. Furthermore, the stock solutions of chlorogenic acid, cichoric acid, luteolin-7-*O*-β-d-glucopyranoside, apigenin-7-*O*-β-d-glucopyranoside and caftaric acid, were prepared in 50% methanol aqueous solution at a final concentration of 20.80, 45.00, 115.00, 104.00 and 95.00 μg/mL, respectively. All the solutions were stored at 4 °C and used to identify the characteristic peaks in the fingerprint of KDZ injection with the exact relative molecular weight and fragment ions.

### 3.3. Chromatographic and Mass Spectrometric Conditions

Fingerprints of the prepared samples were performed on a Waters ACQUITY UPLC CLASS I system (Waters Co., Singapore), equipped with a photo diode array (PDA) detector, quaternary solvent delivery manager, vacuum degasser and auto sampler. Sample separation was achieved on a Waters ACQUITY UPLC^®^ BEH C18 column (100 mm × 2.1 mm, 1.7 μm) with a constant flow rate of 0.2 mL/min at 45 °C. The mobile phase consisted of 0.1% formic acid water (A) and 0.1% formic acid acetonitrile (B), using a gradient elution of 1% B at 0–8 min, 1–4% B at 8–9 min, 4–6% B at 9–15 min, 6–9% B at 15–16 min, 90–11% B at 16–20 min, 11–14% B at 20–21 min, 14–16% B at 21–31 min, 16–22% B at 31–40 min, 22–1% B at 40–42 min, 1% B at 42–45 min. The injected volume was set at 5 μL, and the detection wavelength was 260 nm. Fingerprint chromatograms were obtained and processed using Empower software (Waters Co., Milford, CT, USA).

UPLC/Q-TOF-MS analysis of KDZ injection was performed on the analysis system coupled with UPLC and quadrupole time-of flight mass spectrometry. Sample separation was carried out using the above chromatographic condition. The detection was performed on a Waters Xevo G2 Q-Tof mass spectrometer (Waters Co., Manchester, UK) equipped with an electrospray ionization (ESI) source operating in both positive and negative modes. Ultra-high purity helium was used as the collision gas, and high-purity nitrogen was used as the nebulizer gas. The desolvation gas flow rate was 600 L·h^−1^ at 325 °C. The capillary voltage was 3.0 kV, and nebulizer gas pressure was 350 psi. The molecular masses of ions, in the range of 50 to 1000 Da, were accurately determined with leucine-enkephalin (*m*/*z* 556.2771 and 554.2615) in both positive and negative ESI modes. After data were acquired, original data were obtained and processed by MassLyn×4.1 software (Waters Co., USA) to detect and align the peaks, and the constituents were identified with the exact relative molecular weight and fragment ions.

### 3.4. Data Processing

All batches of KDZ injection samples were analyzed in the chromatography system introduced above to record the chromatographic fingerprints. The fingerprint similarity evaluation was performed on the SESCFTCM, developed by the Chinese Pharmacopoeia Committee and calculated based on the cosine method for the fingerprint chromatograms.

ROC curve and binary logistic regressions were applied to optimize characteristics peaks using PASW 18.0 to obtain specific characteristic peaks with more sensitivity and specificity. Then, characteristics peaks were subjected to SVM to establish a statistical prediction model. The SVM model was developed by LIBSVM in Matlab R2010a (Mathworks, Natick, MA, USA). The SVM model used kernels to map from low-dimensional to high-dimensional spaces, and a penalty factor was used to determine the characteristics of the subspace-regulated learning. The confidence and experience risk ratio ranges were determined by the cross-validation method. In the SVM classification process, the computer was trained by the training set before the classification model was established, and the test set was used to determine the accuracy of the model.

## 4. Conclusions

In the present study, a novel and practical “Fingerprint-ROC-SVM” analytical strategy was established to monitor or predict the quality of KDZ injection. On the basis that the fingerprint similarity evaluation could not distinguish between QPs and UPs samples, the novel chemometrics method, combined ROC and SVM, was used in the established analytical strategy. The characteristic peaks in fingerprints were optimized by AUCs value greater than 0.7 in the ROC curve; then, the optimized characteristic peaks were used to establish the SVM prediction model. Based on the results described above, the CV accuracy of the established model reached 97.06%. Compared with PCA and HCA, the superiority of the novel analytical strategy was more prominent, which indicated that it could effectively and accurately monitor or predict the quality of KDZ injection. Furthermore, the HTPs and HIPs samples were used to further verify the feasibility of the novel analytical strategy. In a further search, the prediction accuracies of the analytical strategy reached at least 93.75%, which indicated that the novel analytical strategy had strong practicality and accuracy once again. In conclusion, based on the case study of KDZ injection, the novel and practical “Fingerprint-ROC-SVM” analytical strategy could be further applied to monitor or predict the quality of TCM injections, which can greatly facilitate the development of quality control and enhance the clinical safety of TCM injections.

## Figures and Tables

**Table 1:** The identification of 12 characteristics peaks based on UPLC/Q-TOF-MS.

No.	t_R_ (*min*)	Positive Ion Mode				Negative Ion Mode				Formula	Chemical Name	References
		Obsd (*m*/*z*)	Calcd (*m*/*z*)	Error (*ppm*)	Fragment Ions	Obsd (*m*/*z*)	Calcd (*m*/*z*)	Error (*ppm*)	Fragment Ions			
1	2.69	-	-	-	-	243.0624	243.0617	−2.88	243 (100), 200, 110	C_9_H_12_N_2_O_6_	Uridine	Standard
2	3.81	268.1041	268.1046	−1.86	268, 136 (100)					C_10_H_13_N_5_O_4_	Adenosine	Standard
3	4.42	284.0978	284.0983	−1.76	284, 152 (100), 113	282.0841	282.0838	1.06	282 (100), 150	C_10_H_13_N_5_O_5_	Guanosine	Standard
4	8.19	-	-	-	-	153.0189	153.0188	−0.65	153 (100), 109	C_7_H_6_O_4_	3,4-Dihydroxybenzoic acid	[15]
5	11.92	-	-	-	-	311.0405	311.0403	−0.64	311 (100), 179, 149	C_13_H_12_O_9_	Caffeoyltartaric acid	Standard
6	19.72	-	-	-	-	353.0881	353.0873	−2.27	353, 191 (100), 179	C_16_H_18_O_9_	Chlorogenic acid	Standard
7	24.87	611.1594	611.1612	−2.95	611 (100), 449, 287	609.1471	609.1456	2.46	609 (100)	C_27_H_30_O_16_	Luteolin-7-*O*-β-d-gentiobioside	[16]
8	25.68	-	-	-	-	473.0739	473.0720	−4.02	473, 311 (100), 293	C_22_H_18_O_12_	Chicory acid	Standard
9	27.91	463.0887	463.0877	2.16	463 (100), 287	461.0724	461.0720	0.87	461 (100), 447	C_21_H_18_O_12_	Luteolin-7-*O*-glucuronide	Standard
10	31.8	423.1667	423.1655	2.84	423, 356, 261 (100)	421.1510	421.1499	−2.61	421 (100), 259	C_21_H_26_O_9_	Ixerin Z	[16]
11	33.14	447.0940	447.0927	2.91	447 (100), 271	445.0787	445.0771	−3.59	445 (100), 425, 259	C_21_H_18_O_11_	Apigenin-7-*O*-glucuronide	Standard
12	34.28	425.1808	425.1812	−0.94	425, 263 (100)	423.1663	423.1655	−1.89	423 (100), 261	C_21_H_28_O_9_	11,13α-dihydroixerin Z	[17]

## Supplementary Materials

The following are available online, Supplementary File 1: Optimization of chromatographic conditions and method validation of fingerprint analysis. Supplementary File 2: Table S1 The similarity between different groups of KDZ injection samples with the reference fingerprint; Figure S1 UPLC fingerprints of 25 batches of UPs after peak alignment; Figure S2 Dendrogram of the clustering of KDZ injection. (A) The QPs vs UPs group; (B) The QPs vs HTPs group; (C) The QPs vs HIPs group; Figure S3 UPLC fingerprints of 25 batches of HTPs after peak alignment; Figure S4 UPLC fingerprints of 25 batches of HIPs after peak alignment.
