# Supplementary material for: A Novel and Practical Chromatographic “Fingerprint-ROC-SVM” Strategy Applied to Quality Analysis of Traditional Chinese Medicine Injections: Using KuDieZi Injection as a Case Study
Source: Molecules. 2017 Jul 23;22(7):1237. doi: 10.3390/molecules22071237 (PMC6152141; doi:10.3390/molecules22071237)
Supplement: Supplementary file 1 [file molecules-22-01237-s001.zip › Supplementary File 2.pdf]

Table S1

Table S1

The similarity between different groups of KDZ injection samples with the reference fingerprint

Batches NO.	QPs	UPs	HTPs	HIPs
**1**	0.998	0.986	0.988	0.994
**2**	0.997	0.994	0.988	0.995
**3**	0.997	0.995	0.991	0.995
**4**	0.999	0.996	0.992	0.995
**5**	0.999	0.995	0.992	0.995
**6**	0.999	0.994	0.991	0.994
**7**	1.000	0.994	0.997	0.997
**8**	1.000	0.996	0.998	0.999
**9**	1.000	0.990	0.997	0.998
**0**	1.000	0.996	0.997	0.998
**11**	0.999	0.990	0.998	0.999
**12**	0.998	0.993	0.999	0.999
**13**	0.999	0.997	0.997	0.998
**14**	1.000	0.996	0.995	0.997
**15**	1.000	0.995	0.994	0.996
**16**	1.000	0.996	0.994	0.996
**17**	0.999	0.986	0.993	0.994
**18**	1.000	0.998	0.996	0.997
**19**	1.000	0.997	0.996	0.998
**20**	0.997	0.994	0.999	0.999
**21**	0.996	0.985	0.999	0.999
**22**	0.999	0.994	0.994	0.996
**23**	0.999	0.992	0.992	0.993
**24**	0.999	0.993	0.993	0.995
**25**	0.999	0.994	0.999	0.997

Fig.S1

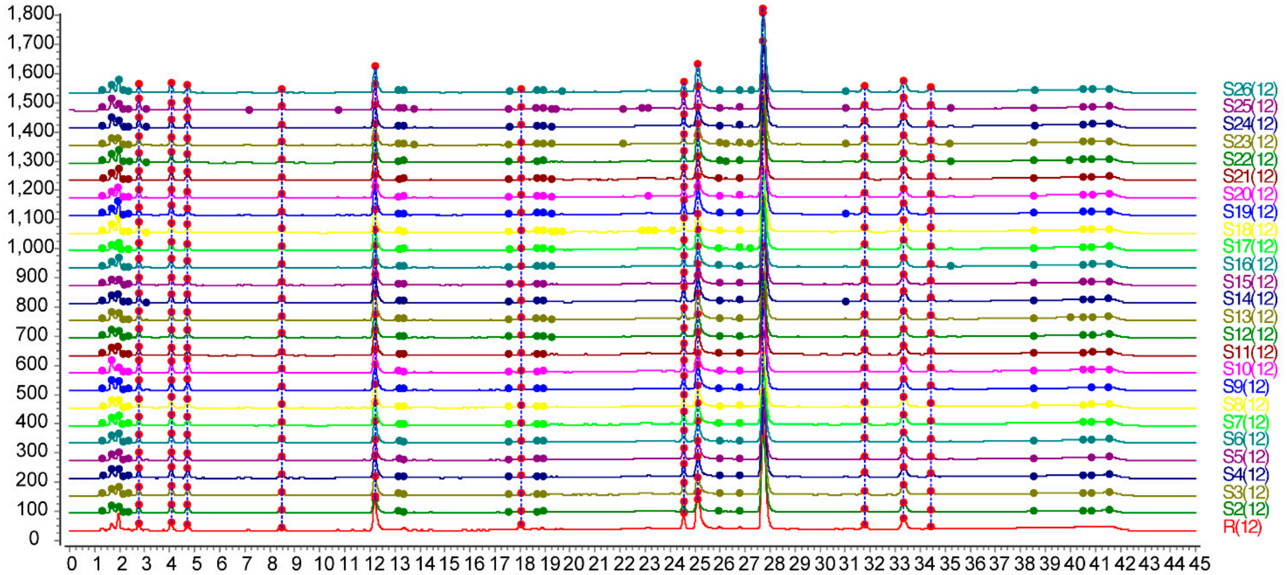

Fig.S1 UPLC fingerprints of 25 batches of UPs after peak alignment

Fig.S2

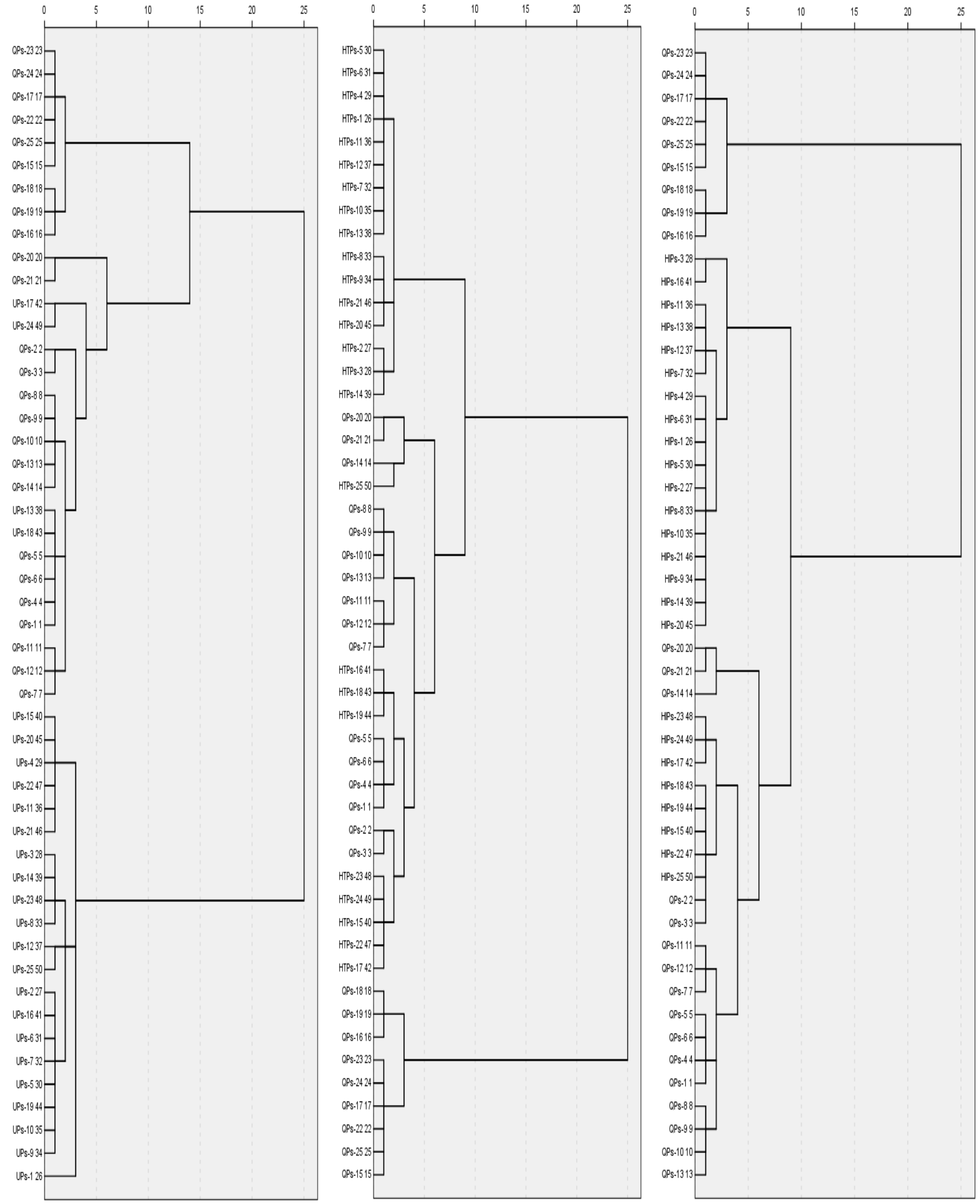

A B C

Fig.S2 Dendrogram of the clustering of KDZ injection. (A) The QPs vs UPs group; (B) The QPs vs HTPs group; (C) The QPs vs HIPs group;

Fig.S3

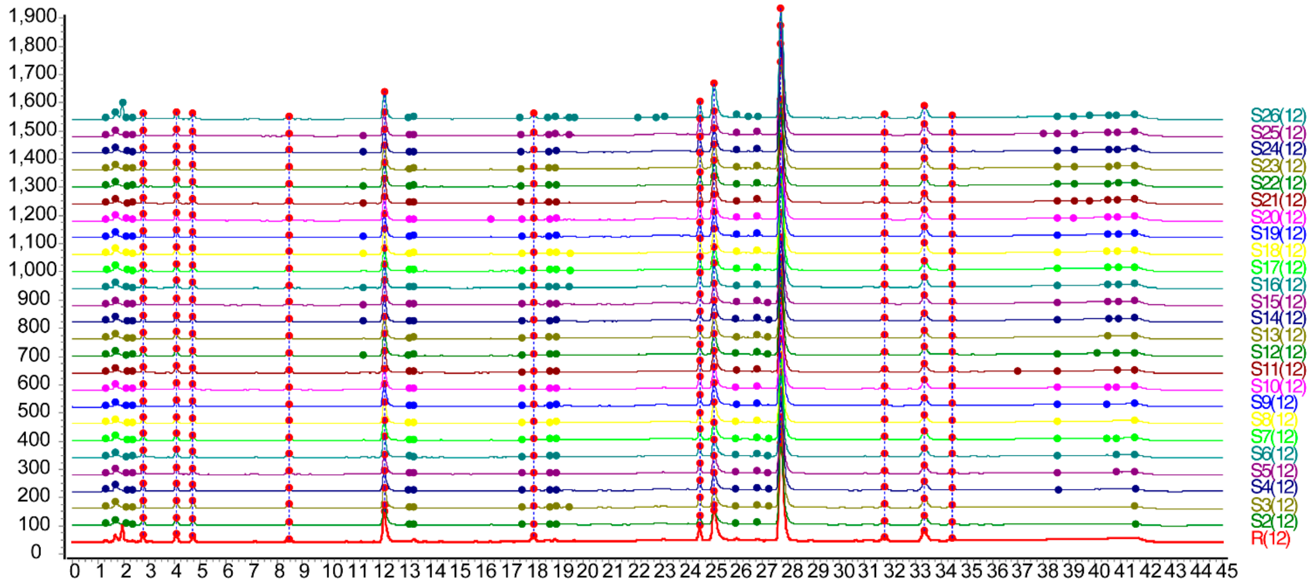

Fig.S3 UPLC fingerprints of 25 batches of HTPs after peak alignment

Fig.S4

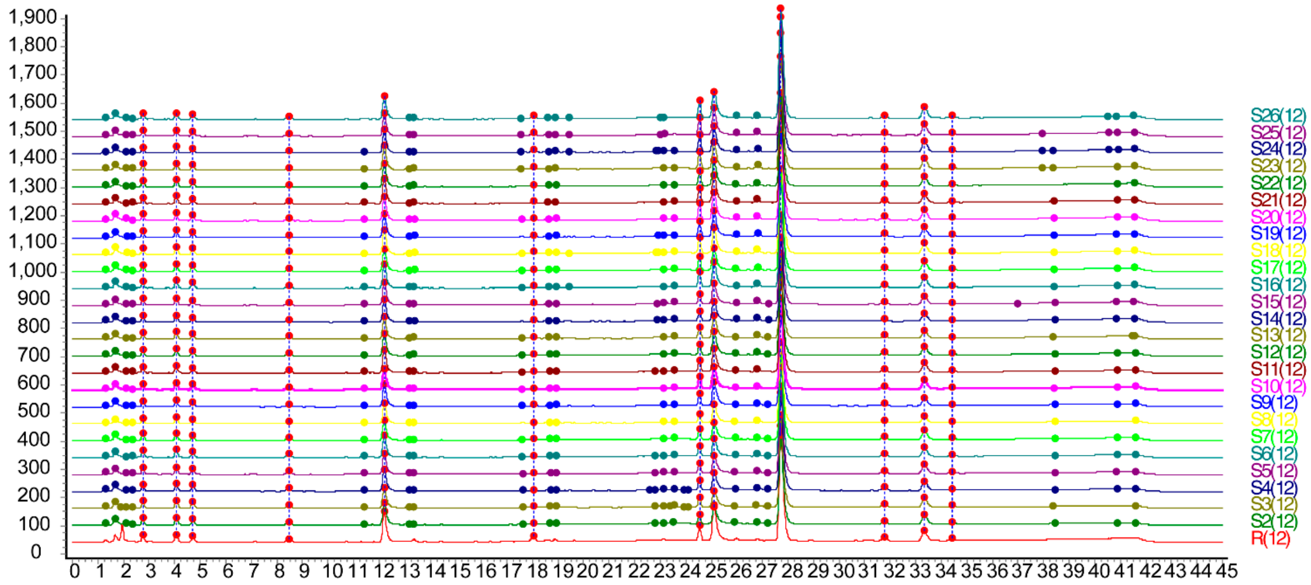

Fig.S4 UPLC fingerprints of 25 batches of HIPs after peak alignment
