# Supplementary material for: A Novel and Practical Chromatographic “Fingerprint-ROC-SVM” Strategy Applied to Quality Analysis of Traditional Chinese Medicine Injections: Using KuDieZi Injection as a Case Study
Source: Molecules. 2017 Jul 23;22(7):1237. doi: 10.3390/molecules22071237 (PMC6152141; doi:10.3390/molecules22071237)
Supplement: Supplementary file 1 [file molecules-22-01237-s001.zip › Supplementary File 1.pdf]

Optimization of chromatographic conditions

The UPLC chromatographic conditions were optimized to obtain a wealth of chromatographic information and good separation effect of the fingerprint. In the optimization, two kinds of mobile phase consist of 1 % GAA-water (A) and ACN (B) and 0.1 % FA-water (A) and 0.1 % FA-ACN (B) were investigated. At the same time, compared 4 kinds of flow rate of 0.4 mL/min, 0.3 mL/ min, 0.25 mL/min and 0.2 mL/min, the results indicated that mobile phase compose of 0.1 % FA-water (A) and 0.1 % FA-ACN (B) at the flow rate of 0.2 mL/min exhibited the highest efficiency of selective separation. In addition, the effects of the amount of sample and column temperature on the chromatographic behavior were studied. The injected volume 2 μL and 5 μL were investigated, respectively. The results showed that flat baseline and obviously peak were observed when the injection volume was 5 μL; Column temperature was investigated at 30, 35, 40 and 45 ℃, respectively, which discovered no significant difference in the chromatographic behavior of column temperature at 35 ℃ and 40 ℃. As the service life of the chromatographic column was considered, the experiments finally performed at 35 ℃.

The chemical constituents in KDZ injection mainly including nucleosides, flavonoids and organic acids. The maximum absorption wavelength of the three kinds of components were different. As the fingerprints should reflect integral chemical characteristics of KDZ injection through a chromatogram, the detection wavelength was required to detect all the three kinds of chemical components. In this optimization, firstly, the sample of KDZ injection was scanned at the wavelength from 210 to 400 nm. Then, the chromatograms under different wavelengths were compared. Eventually, the fingerprint detection wavelength was determined at 260 nm that the three kinds of main components in KDZ injection had obvious absorption.

Method validation of fingerprint analysis

Method precision was determined by analyzing the same sample five consecutive times in one day. The RSD of the relative retention time (RRT) and relative peak area (RPA) of the common peaks were lower than 0.16 % and 2.12 %, respectively.

Repeatability was assessed by analyzing six independently KDZI samples. The RSD of the RRT and RPA of the common peaks were lower than 0.16 % and 0.98 %, respectively.

Sample stability was evaluated using the same samples after 0, 2h, 4h, 8h, 2h, 24h, 36hand 48 h. The RSD of the RRT was less than 0.34 % and the RSD of the RPA was less than 2.71 %. These results indicated that the sample solution was stable within 24 h at room temperature.
